# Deviance sensitivity in the auditory cortex of freely moving rats

**DOI:** 10.1371/journal.pone.0197678

**Published:** 2018-06-06

**Authors:** Ana Polterovich, Maciej M. Jankowski, Israel Nelken

**Affiliations:** 1 Edmond and Lily Safra Center for Brain Sciences, The Hebrew University of Jerusalem, Jerusalem, Israel; 2 The Department of Neuroscience, The Silberman Institute of Life Sciences, The Hebrew University of Jerusalem, Jerusalem, Israel; University of Jyväskylä, FINLAND

## Abstract

Deviance sensitivity is the specific response to a surprising stimulus, one that violates expectations set by the past stimulation stream. In audition, deviance sensitivity is often conflated with stimulus-specific adaptation (SSA), the decrease in responses to a common stimulus that only partially generalizes to other, rare stimuli. SSA is usually measured using oddball sequences, where a common (standard) tone and a rare (deviant) tone are randomly intermixed. However, the larger responses to a tone when deviant does not necessarily represent deviance sensitivity. Deviance sensitivity is commonly tested using a control sequence in which many different tones serve as the standard, eliminating the expectations set by the standard ('deviant among many standards'). When the response to a tone when deviant (against a single standard) is larger than the responses to the same tone in the control sequence, it is concluded that true deviance sensitivity occurs. In primary auditory cortex of anesthetized rats, responses to deviants and to the same tones in the control condition are comparable in size. We recorded local field potentials and multiunit activity from the auditory cortex of awake, freely moving rats, implanted with 32-channel drivable microelectrode arrays and using telemetry. We observed highly significant SSA in the awake state. Moreover, the responses to a tone when deviant were significantly larger than the responses to the same tone in the control condition. These results establish the presence of true deviance sensitivity in primary auditory cortex in awake rats.

## Introduction

The probability-dependent modulation of auditory responses has been extensively investigated, usually using oddball sequences [1]. In such sequences, a common (standard) stimulus and a rare (deviant) stimulus are randomly intermixed. Neural responses to a tone are larger when it is deviant than when it is standard. This finding has been established in many species, at least as early as the inferior colliculus [2], and in particular in primary auditory cortex [1,3–8], and has been named stimulus specific adaptation (SSA). SSA is dependent on the deviant probability, on the dissimilarity between the standard and deviant tones, and on the inter-stimulus intervals [3,9,10]. Moreover, auditory cortex may be sensitive to rules governing the temporal structure of the stimulation sequence beyond stimulus probability: responses in rat auditory cortex were larger when the standard and deviant stimuli were randomly intermixed than when the same stimuli were presented in a periodic pattern [11].

The larger response to a sound when deviant than when standard may reflect a stronger use-dependent fatigue of the neuronal elements driving the responses to the sound when standard [12], so that the contrast between the responses to the standard and deviant sounds is due to the reduction in the responses to the standard, a reduction which does not generalize, or only partially generalizes, to the deviant. In contradistinction, larger responses to a deviant may reflect sensitivity to violations of the expectations set by the standard, indicating a transient memory-based comparison mechanism [13]. Such sensitivity is often called deviance sensitivity [14]. A common test for deviance sensitivity uses a deviant among many standards control sequence, where many different tones serve as the standard, therefore eliminating any expectations about the next stimulus (the deviance of the deviant), while controlling for its rarity (for example Jacobsen and Schroeger[15]). When the response to the tone when deviant against a single standard is larger than the response to the same tone in the control sequence, it is concluded that true deviance sensitivity occurs [14]. When using this control with a frequency interval between nearby frequencies that is the same as that between the standard and deviant tones in the oddball sequence, this control is conservative, since it doesn’t equalize the potential across-frequency adaptation that is often present in the auditory system—the ubiquitous finding that the presentation of one tone frequency reduces responses to later presentations of nearby frequencies as well [9]. However, using smaller frequency intervals in the control sequence than in the oddball sequence mixes up deviance and across-frequency adaptation, making the results hard to interpret [9]. We will refer below to the control condition with frequency intervals that are the same as that between the standard and deviant as the ‘conservative control’.

In human subjects, a widely studied event-related potential called mismatch negativity (MMN) passes the test of deviance sensitivity with the conservative control [14]. MMN and SSA are not identical: MMN is relatively late (more than 100 ms after sound onset) compared with the earliest response components in auditory cortex (starting 20 ms after sound onset in humans, [16]) with which SSA is associated. Indeed, MMN is elicited with complex deviances that fail to evoke SSA [17]. For these and other reasons, MMN is best considered to lie downstream of SSA [4,18–22]. Early cortical responses in humans (mid-latency responses, MLR) have been shown to have SSA [23]. Remarkably, MLR also show deviance sensitivity [24,25].

In primary auditory cortex of anesthetized rats, the responses to deviants and to the same tones in the conservative control condition are comparable in size [9,26,27]. Previously we have interpreted this as evidence for deviance sensitivity: using explicit models of neural fatigue in the feed-forward connections, we have shown that fatigue of the deviant responses in the control condition is essentially always smaller than in the oddball sequence, so that fatigue by itself predicts larger responses in the control than in the deviant condition. Thus, in anesthetized rats the responses to deviants were larger than expected given the responses in the control condition, assuming feedforward fatigue. It is still unknown whether true deviance sensitivity occurs in intracerebral recordings in awake animals, mostly because the appropriate tests have not been used [5–7,10,28–30,27,31]. We review the evidence and justify this statement in the discussion section.

In this study, we explored deviance sensitivity in rat auditory cortex. We used a set of stimulus conditions, employed in previous studies in anesthetized animals [9,26] and in humans [24,32], to discriminate between across frequency adaptation, SSA and true deviance sensitivity. We report the existence of SSA as well as true deviance sensitivity in local field potentials recorded in the primary auditory cortex of awake, freely moving rats.

## Materials and methods

### Preparation

Six adult female Sabra rats weighing 250–380 g were used for this study (Harlan Laboratories). Upon arrival, animals were housed two per cage and handled daily for a week before surgery. They were kept in a temperature and humidity-controlled room and maintained on a 12 hour light/dark cycle (lights on from 07:00 to 19:00). They had free access to water and standard rodent food pellets (Harlan Laboratories) except for the recording sessions. The ethics committee of the Hebrew University approved the study protocol for animal welfare. The Hebrew University is an Association for Assessment and Accreditation of Laboratory Animal Care (AAALAC) International accredited institute.

### Electrodes

Rats were implanted with electrode arrays built of eight tetrodes mounted onto small drivable microdrives (Axona Ltd., UK). Tetrodes were made of one of the following types of insulated wire: Ø25 μm platinum—iridium wire (150–350 kΩ measured when mounted on microdrive), Ø17 μm platinum—iridium wire (250–550 kΩ) or Ø12.5 μm tungsten wire (150–450 kΩ) (California Fine Wire Ltd., CA, USA). The tetrodes were mounted on prepared microdrives (Axona Ltd., UK). In the Microdrive, the tetrodes were supported and separated by polyimide tubes of diameters adjusted to the type of the wire (MicroLumen, Oldsmar, FL, USA). All soldered connections between tetrodes and microdrive wires were insulated and fixed with nail polish to prevent mechanical damage and generation of movement-related electrical noise. Microdrives spanned 0.6 mm-1.9 mm in the rostro-caudal axis at their entry site to the cortex.

### Surgical procedure

**Fig 1:**
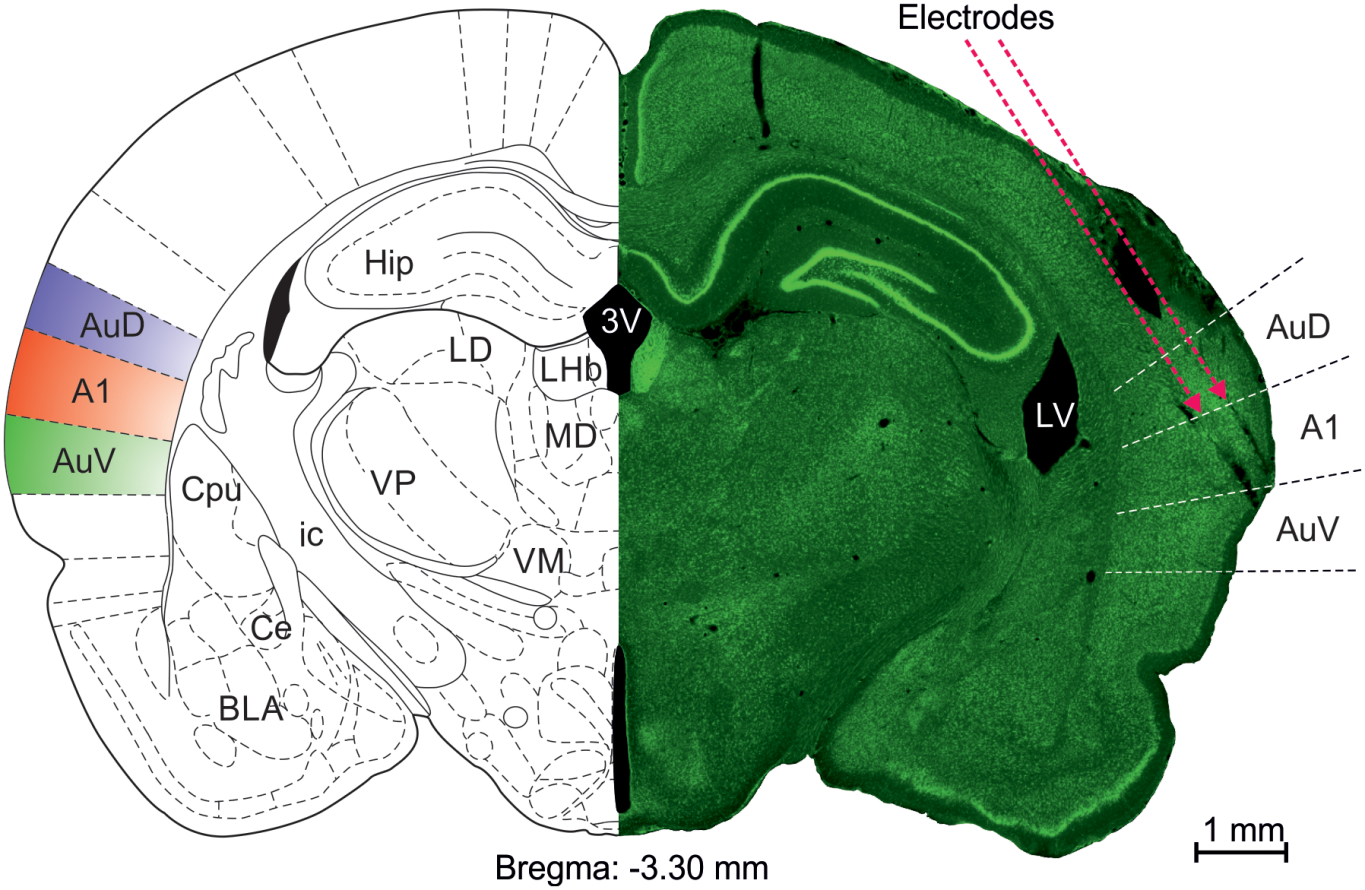
Example slice with electrode track. Right: A fluorescent green Nissl stained coronal section of an example slice (-3.3 mm from bregma). Left side: the corresponding replotted atlas page. Dashed lines denote the borders between auditory areas. Reconstructed direction and angle of electrode paths in pink.

The tetrodes were implanted at the following coordinates targeted at the left primary auditory cortex (AP: 4.3 mm posterior to bregma, lateral position: just medial to the lateral ridge, DV: 2.3–2.4 mm below brain surface). The electrodes were implanted at an angle of about 28 degrees (Fig 1).

Rats were initially anesthetized in an induction chamber with sevoflurane (8% in oxygen, Piramal Critical Care Inc., Bethlehem, PA, USA). Their head was shaved and they were placed in a stereotaxic instrument with a mask for gas anesthesia (David Kopf Instruments, CA, USA). Sevoflurane concentration was slowly adjusted to the level of 2–2.5% and maintained at this level throughout the surgery. Surgical level of anesthesia was verified by lack of pedal-withdrawal reflex. The eyes were protected with a thick layer of Vaseline and the skin on the head was disinfected with povidone-iodine solution (10%, equivalent of 1% iodine, Rekah Pharm. Ind. Ltd., Holon, Israel).

A 1.5–2 cm longitudinal cut of the skin on the head was made and the bones of the skull were exposed. The connective tissue was mechanically removed from the skull and bones were treated with a 15% hydrogen peroxide solution (Sigma Aldrich Inc., St. Louis, MO, USA) which was immediately flushed with sterile saline. When the surface of the skull was clean and dry, a reference point for the entrance of recording electrodes was marked at the chosen coordinates. Subsequently, 7–8 openings for supporting screws were drilled and screws were mounted in the skull. Ground wire was connected to one of the screws placed in the frontal bone. The screws were fixed together and to the bone with dental cement (Coral-fix, Tel Aviv, Israel) forming a base for the implant. The electrode implantation site was kept free of dental cement.

The craniotomy was created by drilling, and the dura gently removed. Tetrodes were slowly inserted into the brain tissue and an external cannula was lowered to create a sealed, telescopic connection making it possible later to lower the electrodes in the microdrive. The microdrive was fixed to the base of dental cement previously prepared on the skull. The ground wire was soldered to the connector of the microdrive and insulated.

The wounds were cleaned and treated in situ with antibiotic ointment (synthomycine, chloramfenicol 5%, Rekah Pharm. Ind. Ltd., Holon, Israel) and Dermatol (bismuthi subgallate, Floris, Kiryat Bialik, Israel). To prevent postoperative pain, rats received subcutaneous injection of Carprofen 50mg/ml (5% W/V) in a dose of about 13 mg/kg (Norocarp, Norbrook Laboratories Limited, Newry, Co. Down, Northern Ireland) immediately following the surgery. Injections of Carprofen were repeated once daily if any symptoms of pain were identified. Rats were allowed at least 1 week of recovery post-surgery. After surgery, animals were housed individually to prevent damage to the implants.

### In-vivo electrophysiology

Recordings were performed using AlphaLab SnR^™^ recording system (Alpha Omega Engineering, Nazareth, Israel) connected with TBSI headstage that included a transmitter for wireless recordings (Triangle BioSystems International, Durham, NC, USA). The 64-channel headstage and the battery (allowing for 10–12 hour recording sessions) were mounted onto custom-made interconnector with a battery holder (total weight of the interconnector with the transmitter and the battery was approximately 15 g). All channels were filtered at 9 kHz and sampled at 22 kHz. Before each recording session, the device was attached to the Axona microdrive on the head of the animal by Mill-Max connectors (MILL-MAX MFG. CORP., New York, USA). The tetrodes were lowered slowly through the brain tissue (at a rate of 25–50 μm/day), typically over a period of weeks, to prevent tissue damage. Based on the daily record of the electrode position and post-mortem histological verification, recording locations could be identified along the recovered electrode traces (Fig 1). We did not control the recordings for wakefulness and sleep.

### Auditory stimulation

Experiments were conducted in a 53x35 cm box (Med Associates, Inc.). Sounds were synthesized online using Matlab (The Mathworks, Inc.), transduced to voltage signals by a sound card (M-16 AD, RME), attenuated (PA5, Tucker Davis Technologies), and played through a stereo power amplifier (SA1, TDT) and a free field speaker (MF1, Tucker Davis Technologies) that was placed above the box. The headstage included a microphone that recorded on one of the channels the sounds that the animals heard. The headstage microphone was calibrated against a B&K microphone (Brüel & Kjær Sound & Vibration Measurement A/S). Sound calibration showed that for pure tones, attenuation level of 0 dB corresponded to 90–100 dB SPL up to 40 kHz and to 80–90 dB SPL for 40–50 kHz.

### Experimental procedure

In each recording session, responses to broad-band noise (BBN) bursts were collected using a sequence of 280 BBN bursts with a duration of 200 ms, 10 ms linear onset and offset ramps, inter-stimulus interval (ISI, onset-to-onset) of 500 ms, and seven different attenuation levels (0–60 dB at 10 dB steps). Levels were presented pseudo-randomly so that each level was presented 40 times. The main data were collected if there was a response to the BBN stimuli at 30 dB attenuation and noise-evoked local field potentials changed regularly with level; otherwise, the tetrodes were lowered by 25–50 μm and recording resumed on the following day.

Responses to tones were collected using quasi-random frequency sequences of 370 pure tone bursts (50 ms, 5 ms rise/fall time; ISI of 500 ms) at 37 frequencies (1–64 kHz, 6 logarithmically spaced frequencies per octave, 10 repeats each). The 370 pure tone bursts were presented at 10 dB attenuation and then again at attenuations decreasing in 10 dB steps until the threshold of the neural activity was reached (usually at 50–60 dB attenuation). These data were used to select the main frequencies and sound levels for the main experiment. Using these responses, the best frequency (BF) was determined as the frequency that gave rise to the most consistent responses at all sound levels. One of the electrodes (usually the one with the best responses) was selected, and two frequencies evoking large responses at 30 dB attenuation (60–70 dB SPL) on either side of the BF of that electrode were selected for further study. The lower frequency was denoted f1, the higher was denoted f2, and they were selected such that f2/f1 = 1.44, corresponding to and interval of 0.526 octave. Responses on all other tetrodes were recorded as well and considered in the final dataset if they passed the inclusion criterion (see Statistics section).

**Fig 2:**
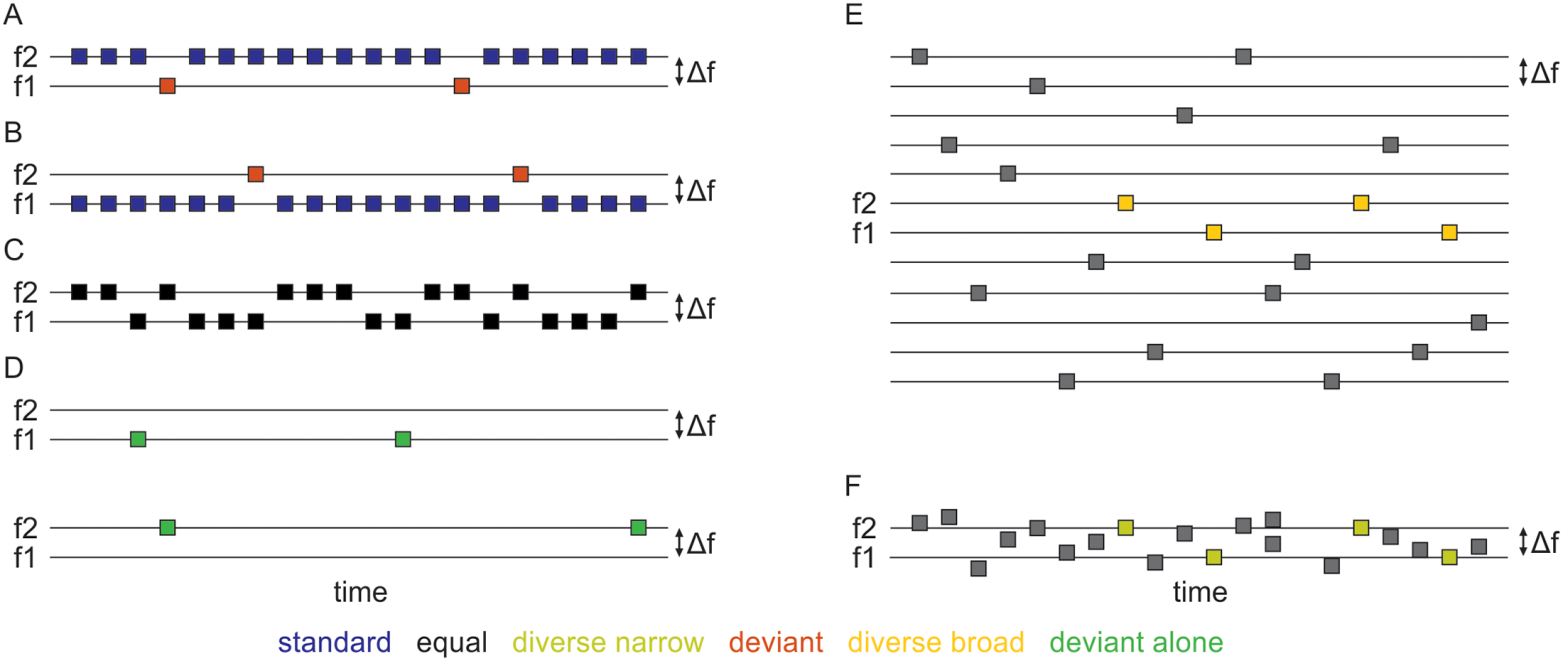
A schematic representation of all test conditions. A. B. and C. Two-tone sequences: in each trial, either f1 or f2 are presented pseudo-randomly according to their probability of occurrence (red, low probability; blue, high probability; black, equal probability). A and B represent the oddball sequences. D. Deviant alone control. Either f1 or f2 appears in 5% of the trials, and the rest is silence. E. Diverse broad control. Frequencies are evenly spaced on a logarithmic scale, with an interval of Δf between each two adjacent frequencies. F. Diverse narrow control. Frequencies are evenly spaced on a logarithmic scale over a range of 2Δf, with f1 the 6^th^ and f2 the 15^th^ frequency in this sequence. Only the responses to the coloured frequencies (f1 and f2) are analyzed here.

We tested the responses to these frequencies in sets of up to seven different sequences, where each sequence consisted of 500 repetitions of 30 ms (5 ms rise/fall time) pure tone beeps with ISI of 300 ms (Fig 2). These sequences have been used previously [9,26].

We presented two oddball sequences: in the first, deviant f1, 475 (95%) of the stimuli presented had frequency f2, and 25 (5%) had frequency f1 (Fig 2A). Here frequency f1 was rarely presented, and therefore denoted deviant, while f2 was commonly presented and denoted standard. In the other oddball sequence, deviant f2, f1 was the standard frequency (95% of the presentations) and f2 was the deviant (5% of the presentations) (Fig 2B). A third sequence, equal, contained equal numbers of f1 and f2 (50%, 250 presentations each), and served as a control for both deviant sequences (see Fig 2C).

Two multi-tone sequences were used. The diverse broad sequence contained tones of frequencies f1 and f2 with probability 5% (25 times each). The other 450 stimuli of the sequence consisted of 10 different frequencies (presented 45 times each, 9% probability). These additional 10 tone frequencies were distributed below f1 and above f2 with successive frequencies separated by the same frequency interval as f1 and f2 (frequency ratio of 1.44, see Fig 2E). Only 12 tones (the two main frequencies and the additional 10 frequencies) were used due to the relatively large Δf, so as not exceed the frequency range of 1–64 kHz. In previous human work [14] as few as 4 different tones in a multi-tone sequence eliminated the deviance of a deviant tone, so that we believe that the asymmetry between the two target frequencies (presented with a probability of 5%) and of the rest (with a probability of 9%) did not result in an unplanned regularity. To the extent possible, frequencies f1 and f2 were positioned at the middle of the frequency range (frequencies no. 6 and 7 among the 12 frequencies, see Fig 2). However, when they were either too high or too low, their location in the frequency range was shifted. For example, for a BF of 20 kHz, the diverse-broad sequence was shifted so that f1 and f2 were the 8^th^ and 9^th^ frequencies in the sequence, there were additional 7 frequencies below f1 and 3 frequencies above f2. In the diverse narrow sequence, tones of frequencies f1 and f2 were played together with 18 other tones, 25 times each. The 20 tones had logarithmically spaced frequencies with the ratio between the lowest and the highest tone in the sequence being 2.16 (slightly more than twice the distance between f1 and f2). These values were selected so that f1 was the 6^th^ and f2 the 15^th^ frequency in this sequence (Fig 2F). Finally, each of f1 and f2 was presented separately in a deviant alone sequence where 5% of the trials had a tone and the others consisted of silence (Fig 2D).

### Histology

After completion of the recordings, rats were placed in the induction chamber with sevoflurane (8% in oxygen) and following induction of deep anesthesia, they received a lethal injection of sodium pentobarbital (900 mg i.p., Pentobarbital sodium 200 mg/ml, CTS Chemical Industries Ltd., Kiryat Malachi, Israel). The rats were then perfused transcardially with 350 ml of 0.1M phosphate-buffered saline (PBS, Sigma Aldrich Inc., St. Louis, MO, USA) at room temperature followed by 400 ml of 4% formaldehyde in 0.1M PBS at ~4°C (Formaldehyde 35%, Bio-Lab Ltd., Jerusalem, Israel). The brains were removed and placed in 4% formaldehyde for at least 72h and transferred to 30% sucrose solution (Sigma Aldrich GmbH, Steinheim, Germany) for about 3–7 days. Brains were blocked, placed on a freezing platform, and 40 μm coronal sections were cut with a sledge microtome (Leica SM 2000R). Brain slices were stained with green fluorescent Nissl stain (NeuroTrace^®^ 500/525 Green Fluorescent Nissl Stain, Molecular Probes^™^, Eugene, OR, USA), mounted onto slides, dried and covered with mounting medium (Vectashield H1200, Vector Laboratories, Inc., Burlingame, CA, USA) and a cover glass. Histological sections were examined using standard fluorescent microscopy.

All slides were analyzed by two independent viewers and their AP location was estimated in reference to the Paxinos and Watson Rat Brain Atlas [33]. Locations of auditory areas A1, AuD and AuV were reconstructed and superimposed with the location of the tips of the tetrodes. Two reconstruction methods were used: one superimposed the size of the slice according to top and bottom distances, and one using the subcortical nuclei. Each recording location (corresponding to one experiment day) was determined according to the reconstruction and assigned to A1 (124 locations), AuD (29 locations) or to AuV (74 locations), ventral to AuV (5 locations) or not classified (129 locations). All electrodes in each location were assigned to the same subdivision. Locations were not classified when the two classification methods did not match. Recording sites in unclassified locations were included in the analysis of the full data, but excluded from the region-specific analyses. In total, 873 electrode sites are analyzed here. Fig 1 shows an example of a histological reconstruction.

To confirm further the recording locations, we used sequences of BFs determined from MUA recordings for which reliable tone responses were present. Sufficient data for the analysis was present for tracks in 4 out of the 6 rats. The BF and depth for each tetrode with a sequence of reliable MUA recordings was compared to the tonotopic organization maps depicted in Polley et al. [34]. All four rats had tetrodes localized to high-frequency A1 dorsally, with the sequence continuing into the high frequency representation at the VAF/AAF border. Some BF sequences could be continued deeper, to SRAF (as the electrodes were lowered on the dorsoventral axis). In two of the rats, we identified anterior electrodes with lower BFs. We interpret these as recording in the middle frequency representation of AAF, then from areas ventral to AAF. In all four rats, recordings localized to A1 by the tonotopic sequences corresponded to histologically identified A1 locations, and recordings located in VAF and SRAF by the tonotopic sequences corresponded to histologically identified AuV locations.

### Data analysis

All data were analyzed with Matlab (Mathworks, Inc., Natick, MA, USA). To detect multiunit activity (MUA), the raw signals were filtered between 200 and 6,000 Hz (50 dB stop bands were at 129 and 6080 Hz) and large, fast events were marked as spikes. The threshold for spike detection was set for each electrode separately to seven times the median of absolute deviations from the median (MAD) of the filtered voltage, corresponding to about 5.5 standard deviations in the case of Gaussian signals. The MUA spike trains were binned into 1 ms bins and the mean spike number in the interval from sound onset up until 50 ms post sound onset was used to quantify the responses. LFP was extracted from the recorded signals by lowpass filtering (corner frequency: 500 Hz, 50 dB stop band 537 Hz) and then downsampling from 22 to 1 kHz. Trials were baseline corrected to the 30 ms interval before stimulus onset. Response strength was quantified by the average response in the interval 10–25 ms after stimulus onset.

### Statistics

All LFP responses to BBN were analyzed, averaged over all contacts within each tetrode (n = 361 recording locations that correspond to 2888 tetrode sites). For further analysis, only recording locations with significant responses to pure tones were selected (n = 196 recording locations that correspond to 1568 tetrode sites). FRAs for LFP signals were also averaged over all contacts within each tetrode. Data selection at the tetrode level reduced the number of non-responding sites being selected for analysis because of statistical fluctuations.

Oddball and control sequences were presented and analyzed for each individual electrode in 111 of these 196 recording locations (n = 3441 individual electrode sites). The main inclusion criterion for data (LFP and MUA) was the presence of significant responses in an electrode site to the deviant alone condition for at least one of the frequencies in each pair. Significance test was performed by a paired t-test (p < 0.01) between the set of single-trial responses and the corresponding pre-stimulus activity levels. Out of 873 significant LFP responses, 364 electrode sites were located in A1 and 285 electrode sites were located in AuV, while 224 electrode sites remained unclassified. Out of 70 significant MUA responses, 29 electrode sites were located in A1 and 21 electrode sites were located in AuV, while 20 electrode sites remained unclassified. No recordings assigned to AuD had significant responses to pure tones. Comparison between all conditions was performed by using linear mixed effects models (Matlab function fitlme), followed by pairwise F tests for the relevant coefficients. This procedure was acceptable because of the very high significance level of the all-condition test. We report p-values, but all p < 10^−20^ will be denoted by 0.

## Results

We presented sound stimuli through a free-field speaker to awake, freely-moving rats (n = 6) and recorded local field potentials (LFP) and multi-unit activity (MUA) from left auditory cortex using 8 movable tetrodes.

### Characterizing the responses

**Fig 3:**
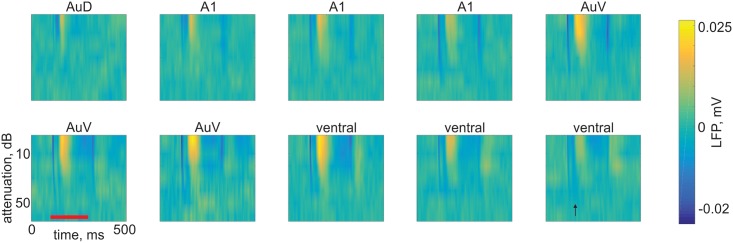
LFP responses to BBN bursts along one electrode track. The responses show gradual changes along the track (from left to right and from top to bottom). Recording locations are about 500 μm apart in A1 and AuV; recording location in AuD is 1500 μm above the first recording in A1; recordings ventral to AuV are about 150 μm apart. Titles were determined from the anatomical reconstruction of the track, from dorsal to ventral. Red line denotes stimulus time. Thresholds tended to decrease as the electrodes entered A1, then increased again when leaving AuV (indicated as ‘ventral’). A second excitatory response occurred sometimes when the electrodes were outside A1 (e.g. rightmost ventral recording location, the most ventral location shown here, marked by a black arrow). The data and code for generating this figure can be found in S1 Data and S1 Code respectively.

Responses to a sequence of broadband noise (BBN) bursts with varying attenuations (0-60dB) were collected at the beginning of each recording session. Fig 3 depicts a typical sequence of responses along one electrode track. We typically observed auditory responses over 3–4 mm along the tracks. LFP onset responses peaked at 18±3 ms (mean±std, n = 2888 tetrode sites) after sound onset. In recording sites dorsal to A1 and in some cases also ventral to A1, a second, later response was observed, between 30 and 60 ms after sound onset (42±4 ms, n = 267; e.g. Fig 3, rightmost ‘ventral’ panel). Offset responses were observed in most recording sites. LFP peak offset responses occurred 29±8 ms (n = 2693) after sound offset.

**Fig 4:**
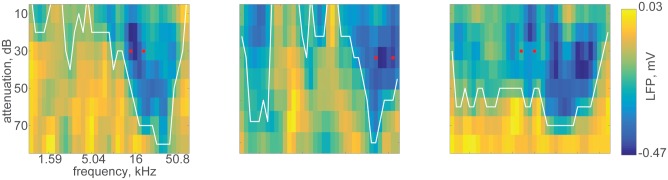
Examples of frequency response areas (FRAs) derived from LFP responses. These were recorded in three different rats. White lines are the outlines of the FRAs. Red dots indicate the frequencies selected as f1 and f2 for the rest of the recording session. The data and code for generating this figure can be found in S2 Data and S2 Code respectively.

Next, responses to pure tone sequences spanning the range of 1–64 kHz were collected. LFP responses to pure tones were typically present to tones at levels as low as 30–40 dB SPL (35 ± 10.5 dB mean±std, n = 1568 tetrode sites). Best frequencies (BF) ranged between 1.5 kHz and 60 kHz, with most of the tetrode sites around 30 kHz, presumably due to the somewhat anterior recording locations. Fig 4 shows examples of a frequency response area (FRA) for LFP recordings from three different electrode sites. Based on an online evaluation of the responses, two frequencies evoking large responses in the best responding electrodes were selected for further study. The red dots in each panel of Fig 4 mark these two frequencies. While many of the FRAs had a typical V shape (Fig 4, left panel), there were also multimodal FRAs with more than one BF (Fig 4, middle panel) as well as broadly-tuned ones (Fig 4, right panel). We did not study FRA shapes in detail.

To analyze the main data for the current study, we used a linear mixed effects model to compare all sequence conditions. In the model, sequence type was a fixed factor (whose levels were standard, deviant, equal, diverse broad, diverse narrow and deviant alone) while animal, experiment day, and electrode site were random factors. Frequencies were considered as repetitions. Sequence type had a highly significant main effect (F(5,8886) = 1520, p = 0 for LFP; F(5,750) = 46, p = 0 for MUA), showing that responses to the different sequence types differed substantially. From now on, we report post-hoc pairwise comparisons using F-tests for the specific contrasts of interest.

### Deviants evoke larger responses than standards

**Fig 5:**
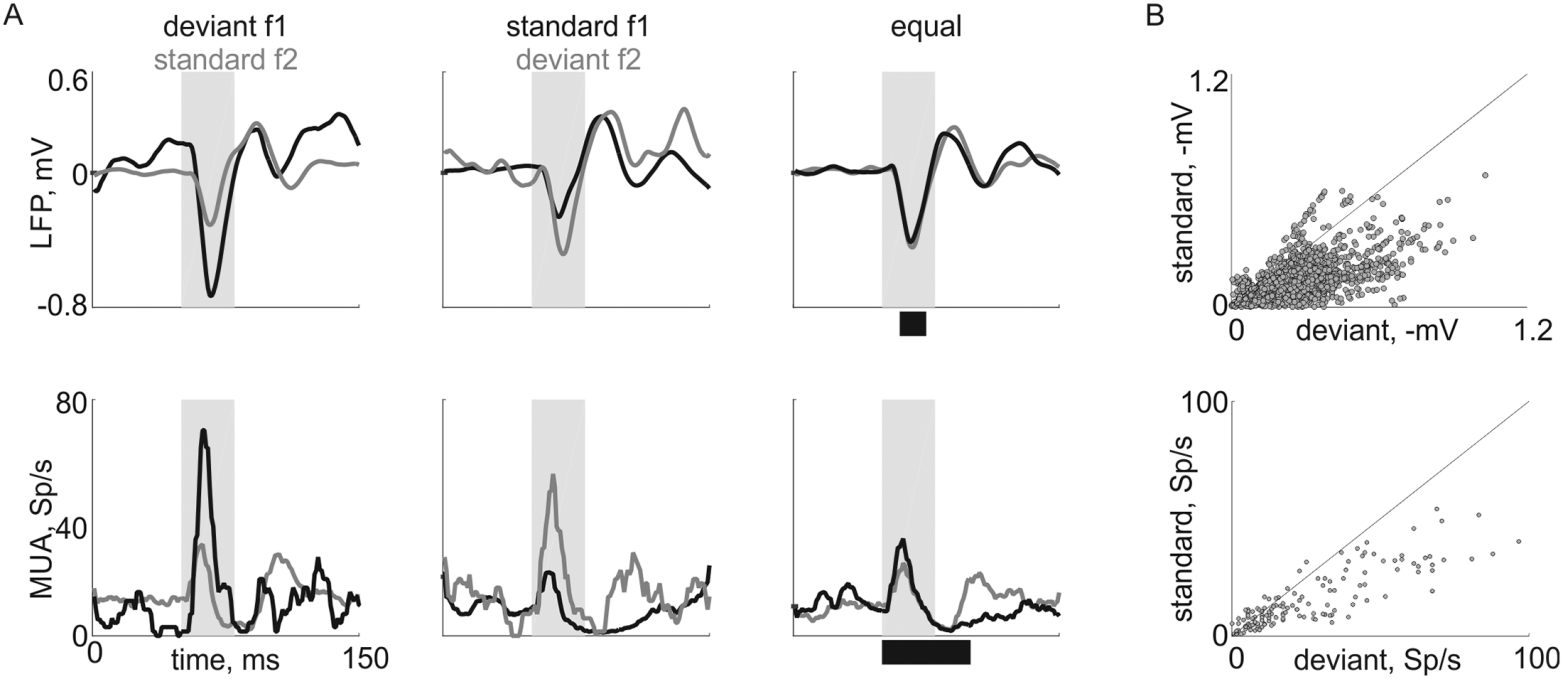
Responses to the two tones in different probabilities of appearance. A. The average LFP (top) and MUA (bottom) responses to the two frequencies (f1 = 37.5 kHz in black, f2 = 54 kHz in grey) in the oddball conditions and the equal condition (see Fig 2A–2C) in one, typical recording site. Tone level was at 30 dB attenuation (~70 dB SPL). Shaded areas: stimulus. Black lines under rightmost panels: the response window. B. Scatter plot of the responses to the same tone when standard and when deviant in all recording sites for both f1 and f2. LFP (top) and MUA (bottom). Black line is the main diagonal (y = x). The data and code generating this figure can be found in S3 Data and S3 Code for panel A and in S4 Data and S4 Code for panel B.

In order to determine whether a response to a tone depends on its probability of occurrence, we compared responses to frequencies f1 and f2 in the two oddball sequences and in the equal condition (for response quantification, see Methods). Fig 5A shows a typical LFP (top) and MUA (bottom) responses from the same recording site to the three sequences. As observed in anesthetized rats using exactly the same stimulation paradigms, the responses to both frequencies when deviant (e.g. deviant f1, left panels black line) were larger than the responses to the same frequency when standard (e.g. standard f1, middle panel black line). Usually the response to the same tone in the equal condition was intermediate (e.g. equal, black line for f1).

Fig 5B compares the responses to a tone when deviant with the responses to the same tone when standard for all recording sites with significant responses (see Methods; n = 873 electrode sites for LFP, n = 70 electrode sites for MUA). Deviant responses were on average significantly larger than standard responses both for LFP and for MUA (linear mixed effects model as above; contrast between standard and deviant responses, F(1,8892) = 1263, p = 0 for LFP, F(1,756) = 58, p = 6.2*10^−14^ for MUA).

**Fig 6:**
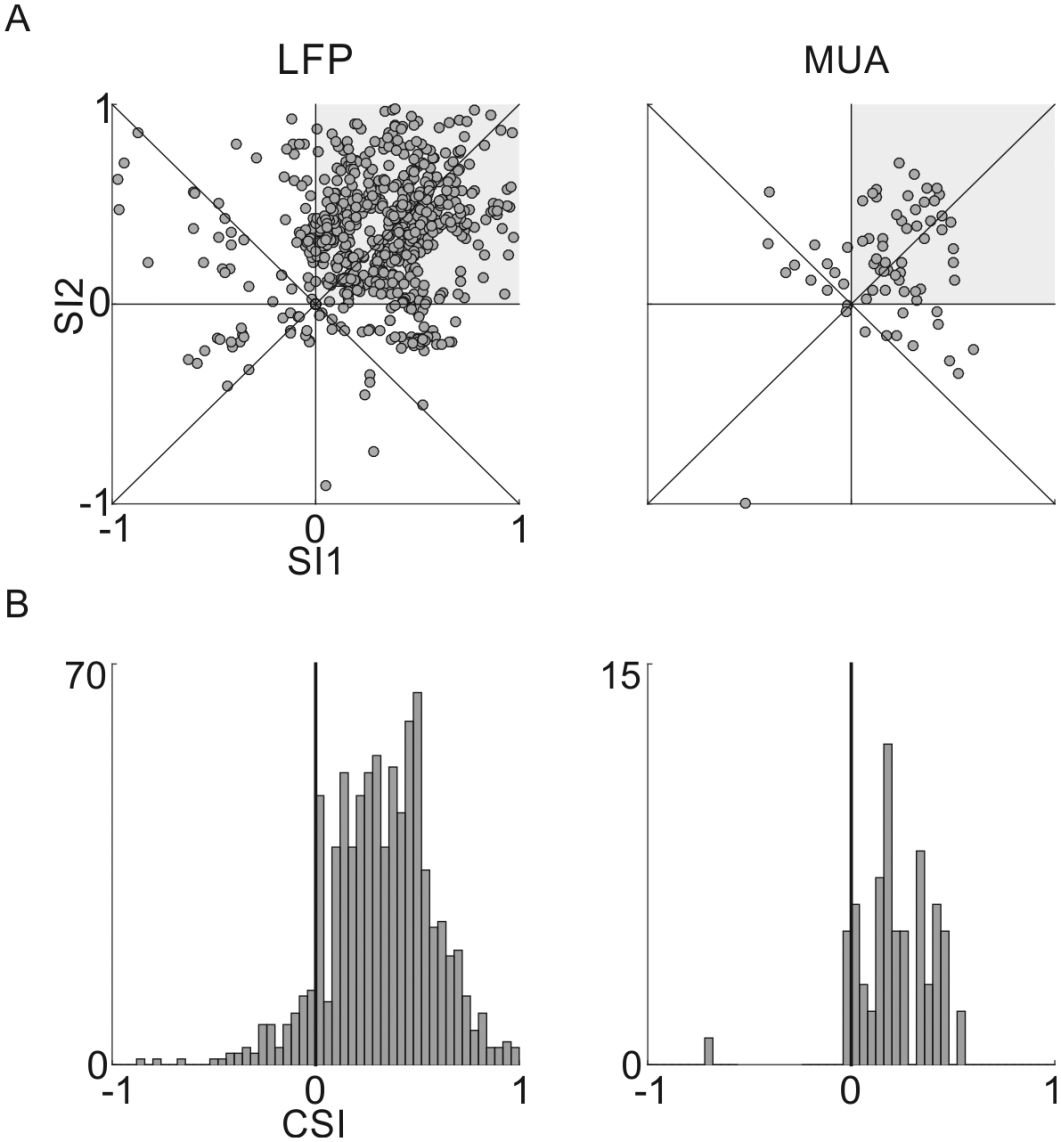
Quantification of SSA. A. SI1 and SI2 plotted against each other for all LFP (left) and MUA (right) electrode sites with significant responses to the tone sequences. Grey area: recording sites that exhibit SSA for both f1 and f2. B. Histograms of the CSIs of all recording sites for LFP (left) and MUA (right). The data and code generating this figure can be found in S4 Data and S5 Code respectively.

The difference between the responses in the standard and deviant conditions was quantified using the SSA Index (SI) [1] (see Fig 6A):

$${SI}_{i}=\frac{d(f_{i})-s(f_{i})}{d(f_{i})+s(f_{i})}$$

Where i = 1,2 for each tone pair, *d*(*f*_*i*_) is the response to the tone when deviant and *s*(*f*_*i*_) the response to a tone when standard. Points in the first quadrant (643/873 for LFP, 46/70 for MUA) indicate that for both *f*_1_ and *f*_2_, the deviant response was larger than the standard response. While there were some cases with negative SI, many of them scattered around the negative diagonal, SI1+SI2 = 0. As shown previously [1], this diagonal is the expected locus of SI1 and SI2 in case of activity-dependent adaptation (‘fatigue’) of the neuronal responses. We also inspected all points in which both SI1 and SI2 were negative. Most of these points, showing anti-SSA (responses to standards that were larger on average than to deviants), occurred in two rats and three experiment days. The anatomical reconstruction localized two of these recordings to AuV and one to A1. Thus, it may be that there are clusters of standard-preferring neurons in these areas, although the paucity of the data precludes any firm conclusion.

The common contrast between the deviant and standard responses was used to characterize the average effect of adaptation for both tones, as defined in previous studies ([9,35], see Fig 6B):

$$CSI=\frac{d(f_{1})+d(f_{2})-s(f_{1})-s(f_{2})}{d(f_{1})+d(f_{2})+s(f_{1})+s(f_{2})}.$$

Mean CSI was 0.31±0.05 for LFPs and 0.21±0.02 for MUA (mean±ste).

**Fig 7:**
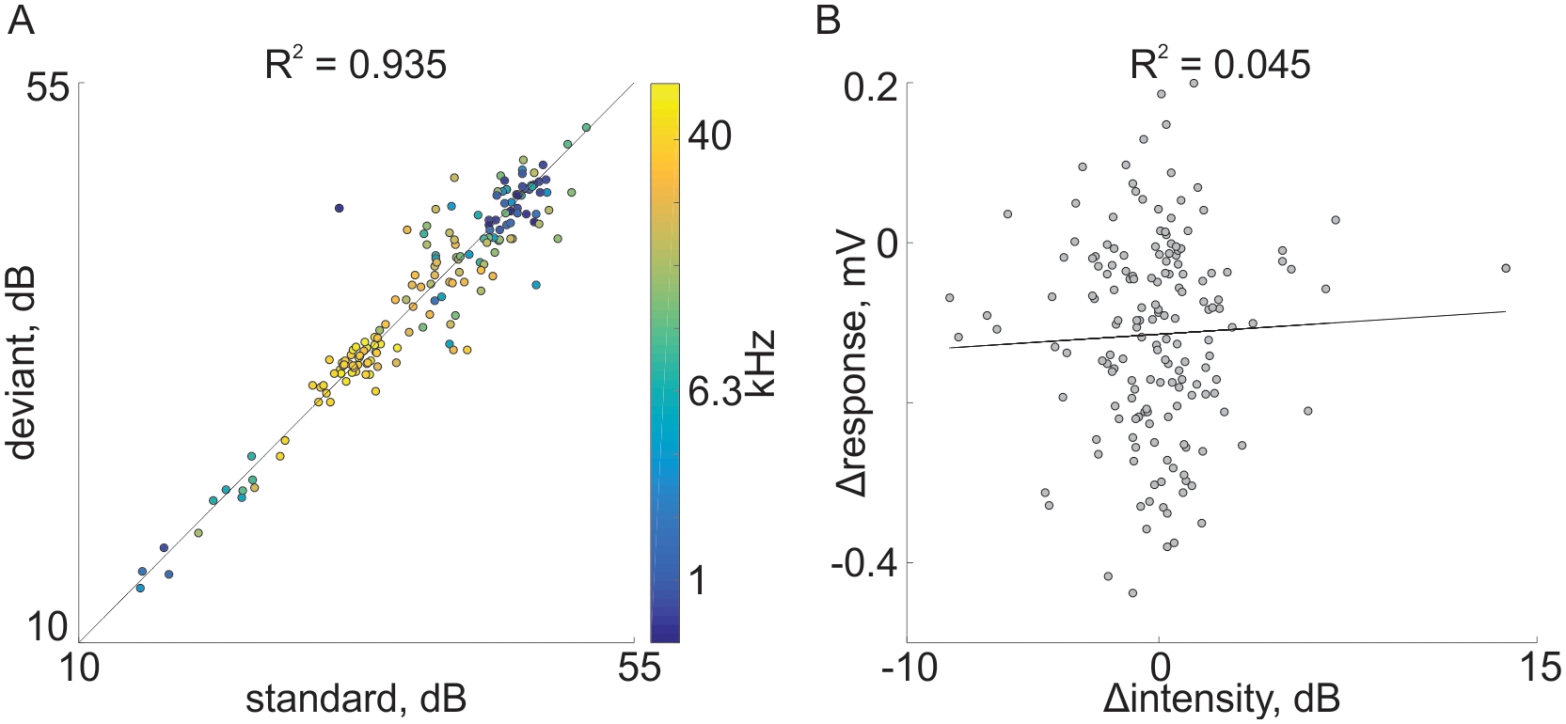
Comparison of sound levels and response sizes. A. A scatter plot of sound levels (determined from the recordings made by the headstage microphone) of a tone when standard (abscissa) and the same tone when deviant (ordinate). Each dot corresponds to one recording session. The colors of the dots correspond to the frequency. Black line is the regression line, with the correlation coefficient displayed above the figure. B. A scatter plot of the difference between the response sizes (Δresponse = response(deviant)–response(standard)) as a function of the difference between the sound levels of the tone in the two conditions (Δintensity = intensity(deviant)–intensity(standard)). Black line is the regression line, with the correlation coefficient displayed above the figure. The data and code generating this figure can be found in S5 Data and S6 Code respectively.

Since the rats were freely-moving and sounds were presented through a fixed loudspeaker, some of the difference in responses to standards and deviants could be due to differences in the actual sound levels heard by the rats. Fig 7 compares the sound levels of the same tones when standard and deviant as recorded by the microphone in the headstage. Each point displays the average sound level computed from all presentations in one recording session in the two conditions. The sound levels for the same tone in the standard and deviant conditions were highly correlated (Fig 7A, R = 0.935) with a slope of 0.947 and an intercept of 1.99, so that they were essentially the same. Moreover, there was no correlation between the difference between responses to standards and deviants and the corresponding difference in sound levels (Fig 7B), consistent with the use of rather high sound levels (e.g. Fig 4) at which response size tends to be saturated. While sound level differences were essentially symmetrical (standard louder than deviant and deviant louder than standard both equally represented), response differences were predominantly negative (standard<deviant). The correlation between the difference in response size and the difference in sound levels was very small (R = 0.045, ns), showing that differences in sound level could not account for the highly consistent SSA.

### Deviants evoke larger responses than diverse broad tones

**Fig 8:**
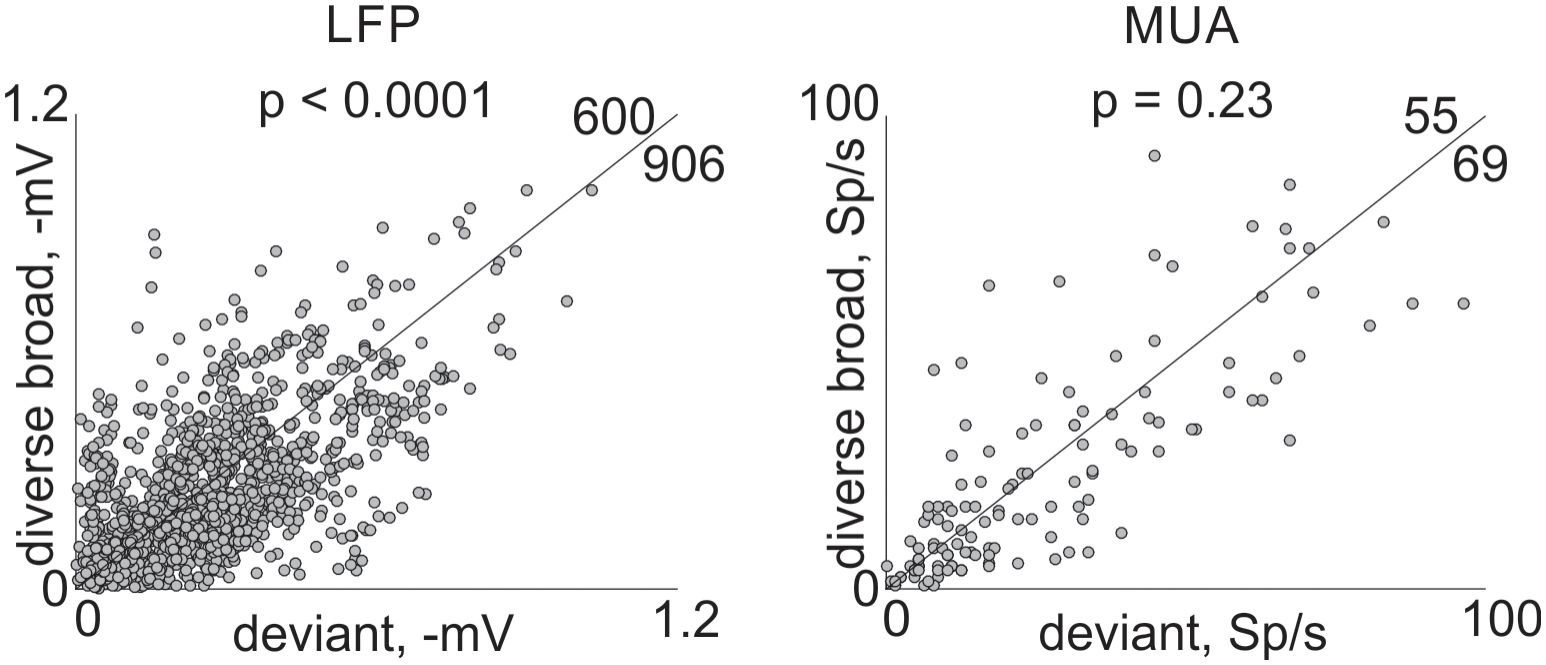
Deviance sensitivity in awake rats. Each dot denotes the responses from a single recording site to a tone when deviant (abscissa) and the response to the same tone in the diverse broad condition (ordinate). LFP (left) and MUA (right) for both f1 and f2. The number of cases on each side of the diagonal are indicated. Black line is the main diagonal (y = x). The data and code generating this figure can be found in S4 Data and S7 Code respectively.

To test true deviance sensitivity, we compared the responses to a tone when deviant and when presented in the diverse broad condition. Fig 8 is a scatter plot of the responses to the same tone in the two conditions. The responses to deviants were significantly larger than to the same tones in the diverse broad condition for LFP (linear mixed effects model as above; contrast between deviant and diverse broad responses, F(1,8892) = 43, p = 3.9*10^−11^) but not for MUA (F(1,756) = 1.4, p = 0.23).

**Fig 9:**
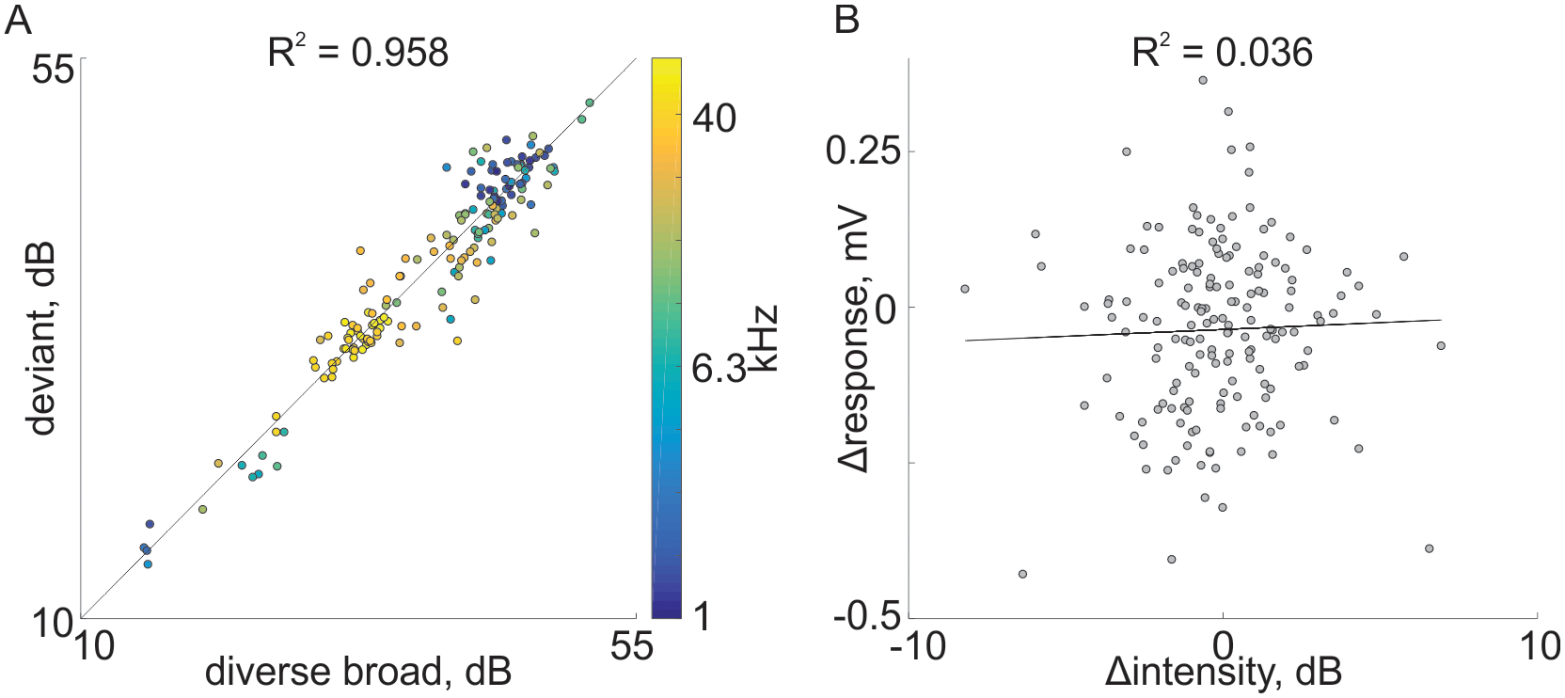
Comparison of sound levels and response sizes. A. A scatter plot of sound levels (determined from the recordings made by the headstage microphone) of a tone in the diverse broad condition (abscissa) and the same tone when deviant (ordinate). Same conventions as in Fig 7. B. A scatter plot of the difference between the response sizes as a function of the difference between the sound levels of the tone in the two conditions. Same conventions as in Fig 7. The data and code generating this figure can be found in S5 Data and S8 Code respectively.

Fig 9 depicts the sound levels of a tone when deviant compared with the same tone when in the diverse broad condition, averaged across all sound presentations used in one recording session. The sound levels for each tone in the two conditions were highly correlated (Fig 9A, R = 0.958) with a slope of 0.983 and an intercept of 0.516, so that they were essentially the same. While sound level differences were essentially symmetrical (diverse broad louder than deviant and deviant louder than diverse broad both equally represented), response differences in the LFP were predominantly negative (diverse broad<deviant). Moreover, there was essentially no correlation between differences in response size and differences in sound level (Fig 9B, R = 0.036, ns). Thus, large differences in sound level were not associated with larger responses to deviant compared to diverse broad.

### Other control sequences

**Fig 10:**
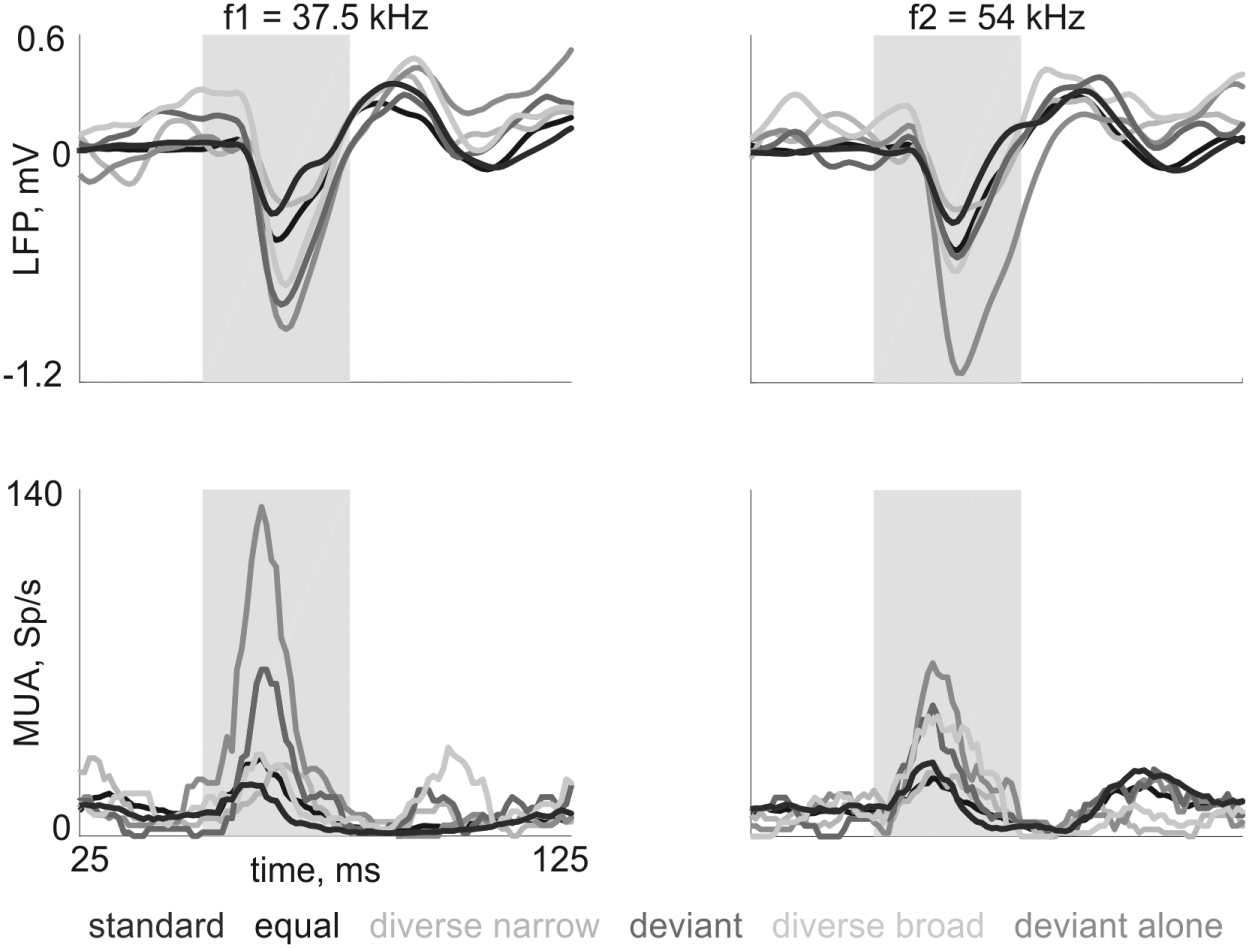
Responses at a typical recording site to all conditions. Example is same as Fig 5. All conditions same as Fig 2. Top: LFP; bottom: MUA. Grey area: stimulus. The data and code generating this figure can be found in S6 Data and S9 Code respectively.

**Fig 11:**
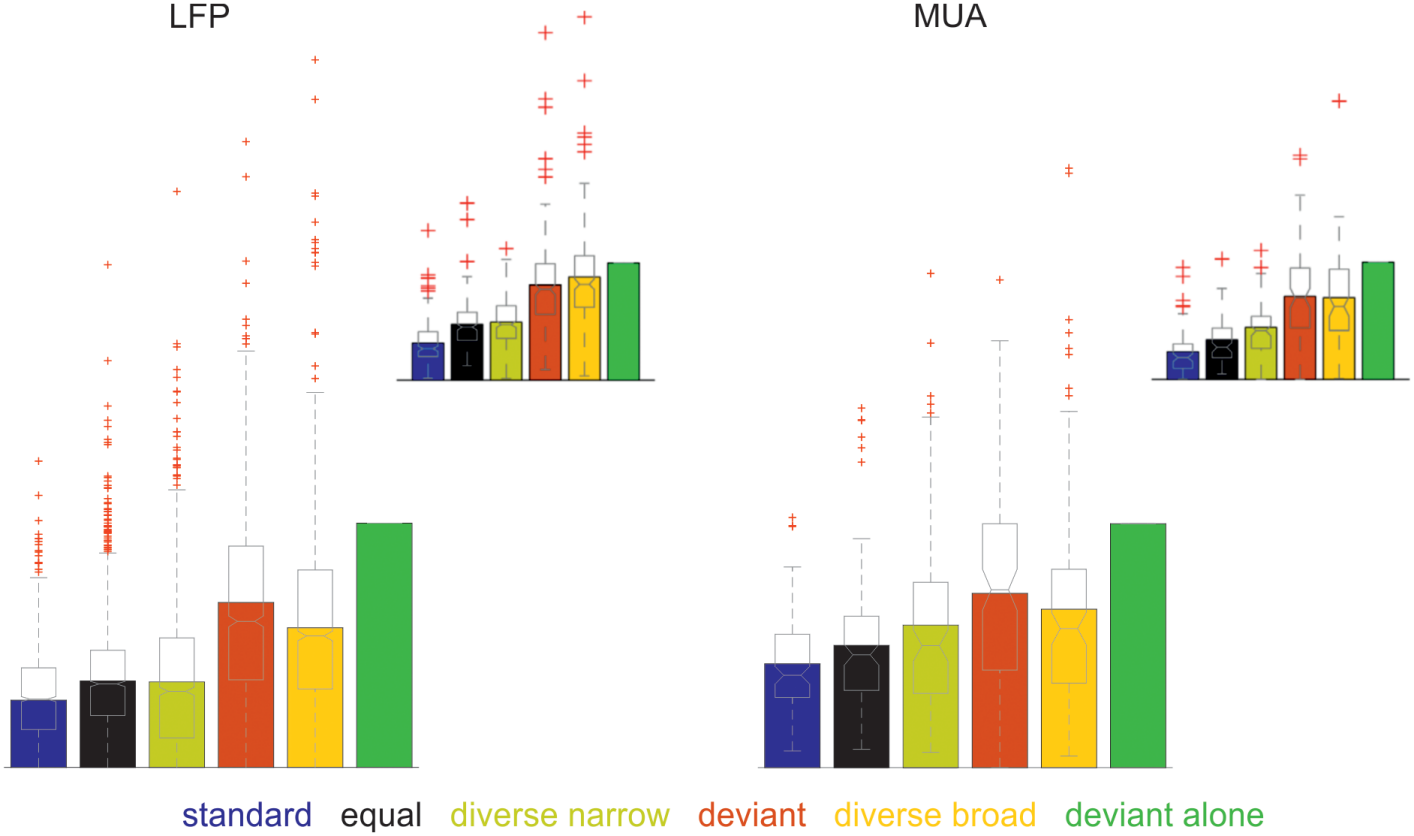
Mean responses to all conditions. Bar plots: mean responses, and box plots: distributions of the responses, normalized with respect to the corresponding deviant-alone response (unadapted response) to both frequencies f1 and f2 in each of the six conditions. Mean responses in all six conditions. Left: LFP; right: MUA. Insets: same data for all six conditions in anesthetized rats, from Taaseh et al. [9]. Conditions are in the same order as in the awake rats. Note the difference between deviant (red) and diverse broad (banana yellow) in the awake compared to the anesthetized conditions. The data and code generating this figure can be found in S4 Data and S10 Code respectively.

Three other control sequences were used: equal, diverse narrow, and deviant alone. These sequences were the same as those used in anesthetized rats by Taaseh et al. [9]. Fig 10 displays typical responses (same recording site as Fig 5). Fig 11 shows the population averages. For both LFP and MUA, tones in the standard condition evoked the smallest responses. Responses were typically small also in the equal condition (presumably because of the still rather high rate of presentations, and therefore adaptation, of the two frequencies) and in the diverse narrow condition where cross-frequency adaptation was presumably strong [9,26]. Typically, responses were larger in the diverse broad and deviant conditions, and largest in the deviant alone condition. The insets in Fig 11 show the average responses recorded in halothane anesthetized rats, using identical tone sequences, replotted from Taaseh et al. [9]. Note the difference in the response to the diverse broad condition, which is larger on average than deviant responses in anesthetized animals (see [9]), but smaller in awake animals (see Fig 8). The relative size of the other conditions was the same in the awake and anesthetized states.

Linear mixed effects models revealed that responses to the deviant alone condition were indeed the largest (contrast between deviant alone condition and the next largest condition—deviant, for LFP: F(1,8892) = 1459, p = 0; for MUA: F(1,756) = 58, p = 9.76*10^−10^; these results are significant after corrections for multiple comparisons). Responses to the equal condition were significantly smaller than the responses to the same tone in the deviant condition (contrast between deviant and equal conditions, for LFP: F(1,8892) = 814, p = 0; for MUA: F(1,756) = 26, p = 3.1*10^−7^). LFP responses to a tone in the diverse narrow condition were significantly smaller compared to responses to the same tone in the diverse broad condition (contrast between diverse broad and diverse narrow conditions, for LFP: F(1,8892) = 854, p = 0; for MUA: F(1,756) = 9, p = 2.7*10^−3^).

### Differences between A1 and AuV

**Fig 12:**
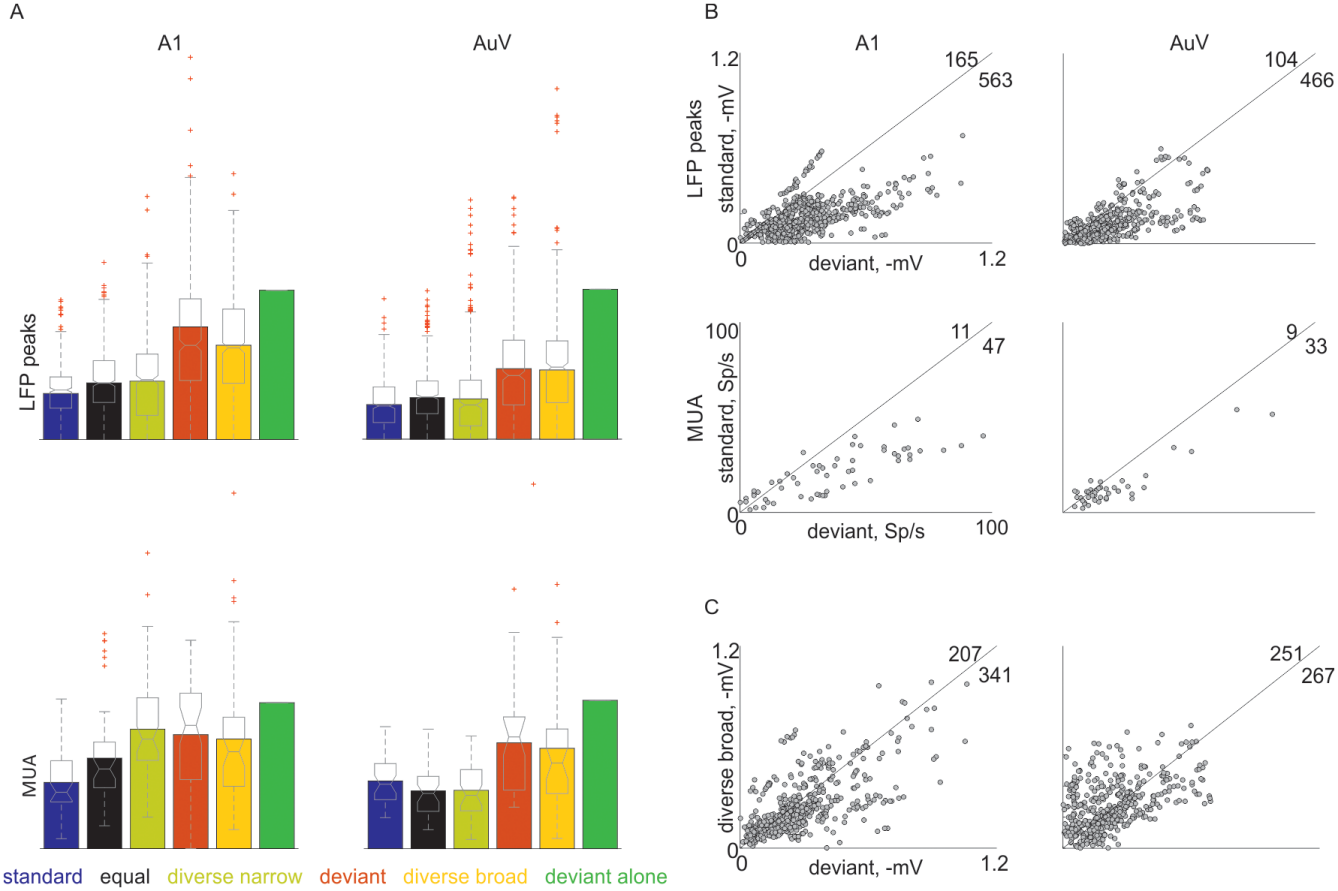
Summary of responses in A1 and AuV. A. Mean responses in all six conditions. Bar plots: mean responses, and box plot: distributions of the responses normalized with respect to the corresponding deviant-alone response (unadapted response) to both frequencies f1 and f2 in each of the six conditions. Left: A1; right: AuV; top: LFP; bottom: MUA. B. Scatter plots of the responses to a tone when deviant (abscissa) compared to the same tone when standard (ordinate) for both f1 and f2. LFP (top) and MUA (bottom) in A1 (left) and AuV (right). Black line is the main diagonal (y = x). The number of cases on each side of the diagonal is indicated in the top right corner. C. Same as B., deviant (abscissa) vs. diverse broad (ordinate) responses for both f1 and f2. Only LFP responses are plotted in C. Black line is the main diagonal (y = x). The number of cases on each side of the diagonal are indicated. The data underlying this figure can be found in S7 Data and the code generating this figure can be found in S11 Code for panel A and in S12 Code for panels B and C.

Recording locations in primary auditory cortex (A1) and ventral auditory area (AuV) were identified using reconstruction of the electrode tracks (see Methods), and responses in both areas were analyzed separately. Overall, responses in A1 were less adapted than in AuV (Fig 12A, linear mixed effects model, fixed factors: condition and auditory area, random factors: animal, and electrode site; main effect of area for LFP: F(1,5320) = 23, p = 1.5*10^−6^; for MUA: F(1,470) = 23, p = 1.9*10^−6^). This is consistent with previous findings [36]. Both areas exhibited SSA both in LFP and MUA (coefficient test; contrast between standard and deviant conditions, A1: F(1,5320) = 355, p = 0 for LFP; F(1,470) = 30, p = 5.2·10^−8^ for MUA. AuV: F(1,5320) = 132, p = 0 for LFP; F(1,470) = 10, p = 0.001 for MUA) (Fig 12B), but SSA (deviant-standard) in the LFP recordings in A1 was significantly larger compared to AuV (coefficient test, F(1,5320) = 23, p = 1.4*10^−6^). Additionally, deviance sensitivity in A1 (the difference between deviant and diverse broad responses) was significantly larger than in AuV (coefficient test, F(1,5320) = 9.5, p = 0.002) (Fig 12A). In fact, deviant and diverse broad responses in AuV were essentially the same on average (Fig 12C).

## Discussion

### Stimulus-specific adaptation in A1 of awake rats

In this study, we examined the responses to oddball and control tone sequences in the auditory cortex of awake, non-behaving, freely-moving rats. We demonstrated the presence of SSA to pure tones both for LFP and MUA signals, in two cortical fields—A1 and AuV. This finding reproduces previous ones in many species, in both anesthetized and awake animals [4,6,7,10,28–30,27,37,38].

In Taaseh et al. [9], neuronal responses to the exact same stimuli were recorded in halothane anesthetized rats. Under the identical stimulation protocols used here, SSA was somewhat smaller in awake than in anesthetized animals. For example, in awake rats, there was a fair number of electrode sites in which at least one of the two frequencies elicited smaller responses when deviant than when standard (negative SI). In Fig 6A, these points lie outside the first quadrant. While not quantified by Taaseh et al. [9], comparing the corresponding plots clearly suggests that such cases were much less common in anesthetized rats. In Fig 6A, many of these points were close the negative diagonal, suggesting the operation of pure adaptation (fatigue, a general activity-dependent reduction in neuronal responses,[1]). Thus, using exactly the same experimental design, we observed more cases of pure, rather than stimulus-specific, adaptation in awake animals. Nevertheless, SSA was found in most recording sites in both awake and anesthetized conditions.

We compared SSA in two auditory areas, A1 and AuV. While SSA was present in both areas, overall adaptation was stronger in AuV than in A1. This result is consistent with previous studies [36]. Remarkably, the contrast between standards and deviants was larger in A1 than in AuV. This is consistent with recent findings in awake mice [27], where SSA was larger in the lemniscal auditory cortex, correlating to A1 in this study, compared to non-lemniscal auditory cortex, corresponding to AuV, due mostly to reduction in the response to the standard condition. One possible confound to this conclusion is the cortical layer where the recordings took place: because of the electrode insertion method (angled relative to the dorsal-ventral axis), recording locations tended to be more superficial ventrally than dorsally. Thus, the difference in SSA strength between A1 and AuV could be due to difference between SSA strength in different cortical layers. Such an account is, however, unlikely. Indeed, Szymanski et al. [39] showed that in rat auditory cortex, SSA tended to be larger in superficial than in the middle cortical layers. Similarly, a recent paper [40] showed that for primary somatosensory cortex, SSA was stronger in more superficial layers (at least for ISIs comparable to those used in Musall et al. [40]). Therefore, if anything, an effect of cortical layer should have increased, rather than decreased, the amount of SSA in our AuV recordings relative to the recordings in A1.

The stronger SSA we found in A1 than in AuV is inconsistent with a recent report of SSA across multiple fields of anesthetized rat cortex [38] who found a progressive increase in SSA from primary to non-primary auditory fields. While their experimental design was somewhat different than ours (tone duration was 75 ms, inter-stimulus intervals 300 ms, 250 trials per block with deviant probability 0.1, 0.5 octaves difference between standard and deviant), it is hard to imagine that these differences caused the discrepancy between the results of Nieto-Diego and Malmierca [38] and ours. We therefore suggest that these differences are due to the use of anesthetized rats by this study [38], and of awake rats by us. Indeed, while responses in A1 under halothane anesthesia are comparable to those in awake animals [41], the urethane anesthesia used by Nieto-Diego and Malmierca [38] produces responses that may differ from the awake condition [42,43]. These anesthesia-related differences may be exacerbated in higher auditory fields. Indeed, the same group reported smaller contrast between standard and deviant in non-lemniscal A1 of awake mice [27].

### Deviance sensitivity in A1 of awake rats

Deviance sensitivity is a stronger property than SSA, and requires additional controls. In the human literature [13,17,24,25,32,44], deviance sensitivity is tested using a condition that corresponds to our diverse-broad control. A number of rodent studies using epidural or scalp recordings reported deviance sensitivity results using control sequences matching ours [31,45]. Our study expands these results by showing deviance sensitivity of intracerebral, local responses. Farley et al. [19] used the same control as our diverse broad sequence and failed to demonstrate deviance sensitivity in MUA recordings. Farley et al. [17] did not report LFP responses. Thus, our results, showing no deviance sensitivity in the MUA recordings, are consistent with theirs.

Other studies in awake animals [4,6,7,10,28–30] implemented control sequences that deviated in important ways from those used in the human literature. For example Nakamura et al. [10], who examined epidural potentials above rat auditory cortex, used Δf = 0.18 for the control sequence, compared to Δf = 0.37 in the oddball condition. Jung et al. [7] studied epidural potentials in rat A1. In the oddball condition, they used narrow band noise stimuli with frequency difference of about 0.58 octave between the highest frequency of the low sound and the lowest frequency of the high sound. In contrast, their control conditions had either overlapping bands or sounds with differences of 0.06–0.11 octave between the high frequency of the lower sound and the low frequency of the higher sound. Similar observations apply to experiments in another animal model: Fishman And Steinschneider [5] explored SSA and deviance detection in awake macaque monkeys, and found that responses to tones in the deviant and control conditions were comparable, claiming no deviance sensitivity. However, they used highly variable Δf both for the oddball and the control sequences, essentially mixing diverse narrow and diverse broad types of control as used here.

Thus, most previous studies used a control condition that was comparable to our diverse narrow control, with small Δfs between adjacent tones or overlapping narrow bands, rather than to the conservative control used in human studies, which corresponds to our diverse broad control. Importantly, in the diverse narrow control, the smaller responses to the target tone are most probably due to across-frequency adaptation triggered by the dense set of frequencies that occur in these blocks, rather than to deviance sensitivity. Both Taaseh et al. [9] and Hershenhoren et al. [26] estimated that across-frequency adaptation occurs within about 1/3 octave in rat A1.

The current results reproduce the results of previous studies in the sense that deviant responses tended to be larger than the responses in the diverse narrow control. Thus, although Nakamura et al. [10], Jung et al. [7] and others reported deviant responses that were larger than control, their results do not imply the presence of true deviance detection in rat A1 because of the confounding across-frequency adaptation. In consequence, their results cannot be used to argue either for or against true deviance detection in primary auditory cortex.

In a recent study, Parras et al. [27] used the conservative control sequence, recording responses in anesthetized rats and awake mice. However, they used in addition highly regular ascending and descending sequences, reporting in awake animals only the responses in the regular condition. Importantly, the regular sequences are expected to show larger adaptation than random sequences both due to their regularity [11] and to across-frequency adaptation [9]. Indeed, Parras et al. [27] found that deviants evoked larger responses on average than the same tone in their regular control sequences even in anesthetized rats; this is definitely different from the use of the fully random control sequence in the same preparation as reported by Taaseh et al. [9] and Hershenhoren et al. [26].

Our study is therefore the first in awake rodents to use a conservative control condition for deviance sensitivity of intracerebral signals that is fully compatible with the control condition used in human studies. Here, we show unambiguously the presence of true deviance sensitivity in the primary auditory cortex (A1) of awake rats: local field potentials evoked by tones in the deviant condition were on average larger than in the diverse broad (control) condition. In AuV, LFP responses to deviants were on average the same in the diverse broad condition. We speculate therefore that the larger SSA in A1 than in AuV in awake rats is a reflection of deviance sensitivity in A1, which boosts specifically responses to deviants. Additionally, we propose that in rats the deviance-sensitive process is sensitive to anesthesia. Indeed, in anesthetized rats, where SSA is larger in AuV than in A1 [38] and where even in A1 diverse broad responses are comparable to deviant responses [9,26], this boost is presumably missing.

## Supporting information

S1 Code Matlab code generating Fig 3. (M)

S2 Code Matlab code generating Fig 4. (M)

S3 Code Matlab code generating Fig 5A. (M)

S4 Code Matlab code generating Fig 5b. (M)

S5 Code Matlab code generating Fig 6. (M)

S6 Code Matlab code generating Fig 7. (M)

S7 Code Matlab code generating Fig 8. (M)

S8 Code Matlab code generating Fig 9. (M)

S9 Code Matlab code generating Fig 10. (M)

S10 Code Matlab code generating Fig 11. (M)

S11 Code Matlab code generating Fig 12A. (M)

S12 Code Matlab code generating Fig 12B. (M)

S1 Data Selected dataset for Fig 3. MAT-file containing BBN examples used in Fig 3.
(MAT)

S2 Data Selected dataset for Fig 4. MAT-file containing FRA LFP examples used in Fig 4.
(MAT)

S3 Data Selected dataset for Fig 5A. MAT-file containing oddball and equal control sequences used in Fig 5A.
(MAT)

S4 Data Selected dataset for Figs 5b, 6, 8 and 11. MAT-file containing dataset of all LFP and MUA responses to oddball and all control sequences.
(MAT)

S5 Data Selected dataset for Figs 7 and 9. MAT-file containing dataset of microphone recordings and responses in corresponding experiment days.
(MAT)

S6 Data Selected dataset for Fig 10. MAT-file containing dataset of an example of LFP and MUA responses to oddball and all control sequences.
(MAT)

S7 Data Selected dataset for Fig 12. MAT-file containing dataset of all LFP and MUA divided into A1 and AuV responses to oddball and all control sequences.
(MAT)
